# Avoiding “shiny objects” and heeding “red flags”: a sensemaking framework for emerging technology procurement

**DOI:** 10.1093/jamiaopen/ooag201

**Published:** 2026-10-12

**Authors:** Wiljeana Glover, Dessislava Pachamanova, Derick R Simmons, Robin Sodano, Eric Alper

**Affiliations:** Kletjian Foundation Distinguished Professor of Health Innovation and Entrepreneurship, Operations and Information Management Division, Babson College, Wellesley, MA 02457, United States; Zwerling Family Endowed Term Chair, Mathematics, Analytics, Science and Technology Division, Babson College, Wellesley, MA 02457, United States; Doctoral Candidate, Saint Louis University, St. Louis, Missouri 63103, United States; Senior Vice President, Information Services, UMass Memorial Health, Worcester, MA 01605, United States; Chief Quality Officer, Chief Clinical Informatics Officer, UMass Memorial Health, Worcester, MA 01605, United States

**Keywords:** technology procurement, emerging technologies, sensemaking

## Abstract

**Objectives:**

Health systems can be vulnerable to “shiny object syndrome,” adopting unneeded emerging technologies (ETs), or may ignore “red flags” if they rely on linear health technology procurement processes that exclude the influence of information exchanges between the health system, vendors, and industry partners.

**Methods:**

We conduct a post-hoc comparison of 2 procured communication-related clinical workflow ETs and examine ET-related IT Committee meetings within an academic medical center. We apply sensemaking theory, highlighting examples where sense-giving, sense-demanding, and sense-breaking were present or absent across stakeholders during procurement.

**Results:**

Present (or absent) sensemaking dynamics demonstrated reciprocity (or lack thereof) across all 3 sensemaking components. Sense-breaking particularly informed committee meetings, leading to new technological and organizational infrastructure.

**Conclusions:**

Our emergent sensemaking ET procurement framework offers 3 actionable recommendations: formalize multi-stakeholder information exchange roles, treat vendor responsiveness to sense-demanding as a selection criterion, and use each procurement cycle to surface and address foundational governance and infrastructure gaps.

## Introduction

As cloud-based and AI-enabled emerging technologies (ETs) proliferate, health systems increasingly struggle to identify solutions that align with organizational needs. In 2022, 703 ETs were compatible with major electronic health records (EHRs) alone,^1^ heightening the risk of “shiny object syndrome,” the implementation of novel technologies misaligned with strategic priorities.^2^ Health systems are also inundated with information from multiple key stakeholders including internal sources, peers, vendors, and consultants;^3–5^ this may obscure the system’s ability to sense “red flags” or technological limitations or vendor capability.^6^ Current procurement process guides are often linear and focus on health system activities;^7^ they exclude the dynamic information exchanges between the health system, potential vendors, and industry partners that influence successful procurement and implementation.

To address these limitations, we examine ET procurement through the theory-informed approach^8–9^ of sensemaking, ie, how organizational actors create and maintain a coherent understanding of new or changing environments to enable action.^10^ Prior research has applied sensemaking to IT teams, health record digitization, clinician responses to decision support, and technology-vendor selection in other industries.^11–14^ To our knowledge, this is the first study to examine the use of sensemaking for healthcare informatics procurement. We present a case report from a large academic medical center (AMC) illustrating sensemaking during both individual ET procurement and enterprise-level procurement strategy decisions.

## Background

Three socio-cognitive components of sensemaking are sense-giving, sense-demanding, and sense-breaking. *Sense-giving* involves efforts to shape others’ understanding, such as justifying or framing a technology.^15–17^  *Sense-demanding* includes efforts to seek clarity via acquiring and processing additional information and thus reducing uncertainty, eg, by seeking peer input or additional documentation from a potential vendor.^15^^,^^14^  *Sense-breaking* includes questioning existing assumptions and reexamining the course of action, for example, by asking why.^15–16^ These 3 components can be present or absent in the communications of health systems, ET vendors, and industry partners, influencing procurement outcomes. Table 1 summarizes these definitions and provides examples of each component.

**Table 1.** Three components of sensemaking and examples of their presence or absence.

Components and their definitions	Presence of component example	Absence of component example
***Sense-giving:* acts to influence the meaning construction of others towards a preferred reality, eg, offering justifications or descriptions to incorporate a technology^15–17^.**	Vendor (or health system) provides details about a solution (or need to be met)	Vendor (or health system) does not provide details or provide limited details about a solution (or need to be met)
***Sense-demanding:* efforts to seek clarity via acquiring and processing additional information to ameliorate current information, thus reducing uncertainty, eg, requesting clarification or cross-checking with peer organizations^14^^,^^15^.**	Health system (or vendor) requests additional information from a vendor (or health system)	Health system (or vendor) does not request additional information, although clarity of existing information was needed
***Sense-breaking:* efforts to reconsider the organization’s existing understanding, eg, via “questioning the bases on which they have been acting” and reexamining the course of action^14–16^**	Health system (or vendor) questions information or receives disruptive cues that that leads to changes in perspective or a previous decision	Health system (or vendor) is not open to questioning or ignores disruptive cues, thus does not change perspective or a previous decision

## Case methods

### Research setting

AMC_North is a large, integrated U.S. AMC with approximately 1200 inpatient beds across four campuses. AMC_North had a formalized ET procurement (ET-P) process with, on average, 120 projects within the planning pipeline in 2024. Their most recently completed projects were application efforts (65%), with the remainder spanning clinical engineering, facilities and capital planning, and infrastructure and cyber security initiatives. Projects were evaluated using standardized criteria: problem statement, solution scope, strategic alignment, risks, EHR integration, project prioritization score, and cost-benefit analysis. AMC_North also leveraged a third-party risk assessment process for each potential ET, requesting compliance and other requirements. Our research team includes 3 researchers affiliated with other academic institutions and 2 hospital leaders from AMC_North. The study was approved by the institutional review board.

### Data collection and analysis

To best capture previous procurement lessons learned as well as current procurement processes, we conducted data collection in 2 phases.

In phase 1, we conducted a post-hoc case comparison of 2 communication-related clinical workflow ETs (Technology 1 (T1)—ambient scribes and Technology 2 (T2)—inter-provider clinical communications, including specialist referrals) that recently went through the AMC_North technology procurement pipeline, comparing a successful vs unsuccessful implementation. We define success via the implementation outcome of the RE-AIM framework for informatics-related interventions, ie, at the setting level, we examine the extent to which the technology was consistently delivered as intended within the specified time and cost.^18^ We chose to focus on communication-based clinical workflow solutions because of their ability to automate portions of the clinical workflow,^19–21^ their rapid health system adoption from 30% to 61% of health systems from 2022 to 2024,^22^ and their varied success.^23^^,^^24^^,^^21^ We used 3 sources of qualitative data:^25^ (1) interviews with 8 healthcare providers and administrators, (2) archival data from the procurement and implementation processes, the Request for Proposal process materials, and the ET product requirement files, and (3) information from the ET organization websites.

In phase 2, we completed direct observations of 3 75-minute AMC_North IT Committee meetings (Quarter 4 of 2024 and Quarters 1 and 2 of 2025). On average, 20 participants joined each meeting, including top organizational leaders (C-suite and VP-level executives) across the Information Management, Information Security, Quality, Clinical Informatics, and Inpatient and Outpatient functions. We also analyzed the presentation files from these meetings. Finally, we interviewed 3 key C-suite executives for additional context and information triangulation. For both phases 1 and 2, we used a directed content analysis approach enabled by NVivo 14. This is appropriate when some theory exists that can inform an initial coding scheme (ie, sense-giving, sense-demanding, and sense-breaking), but when further description of the specific components of interest is still needed (ie, procurement process stages).^26^

## Results

### Phase 1: comparing 2 ETs

Figure 1 summarizes our findings from T1 and T2, highlighting sense-giving, sense-demanding, and sense-breaking activities between AMC_North, vendor 1, vendor 2, and industry partners.

**Figure 1.**
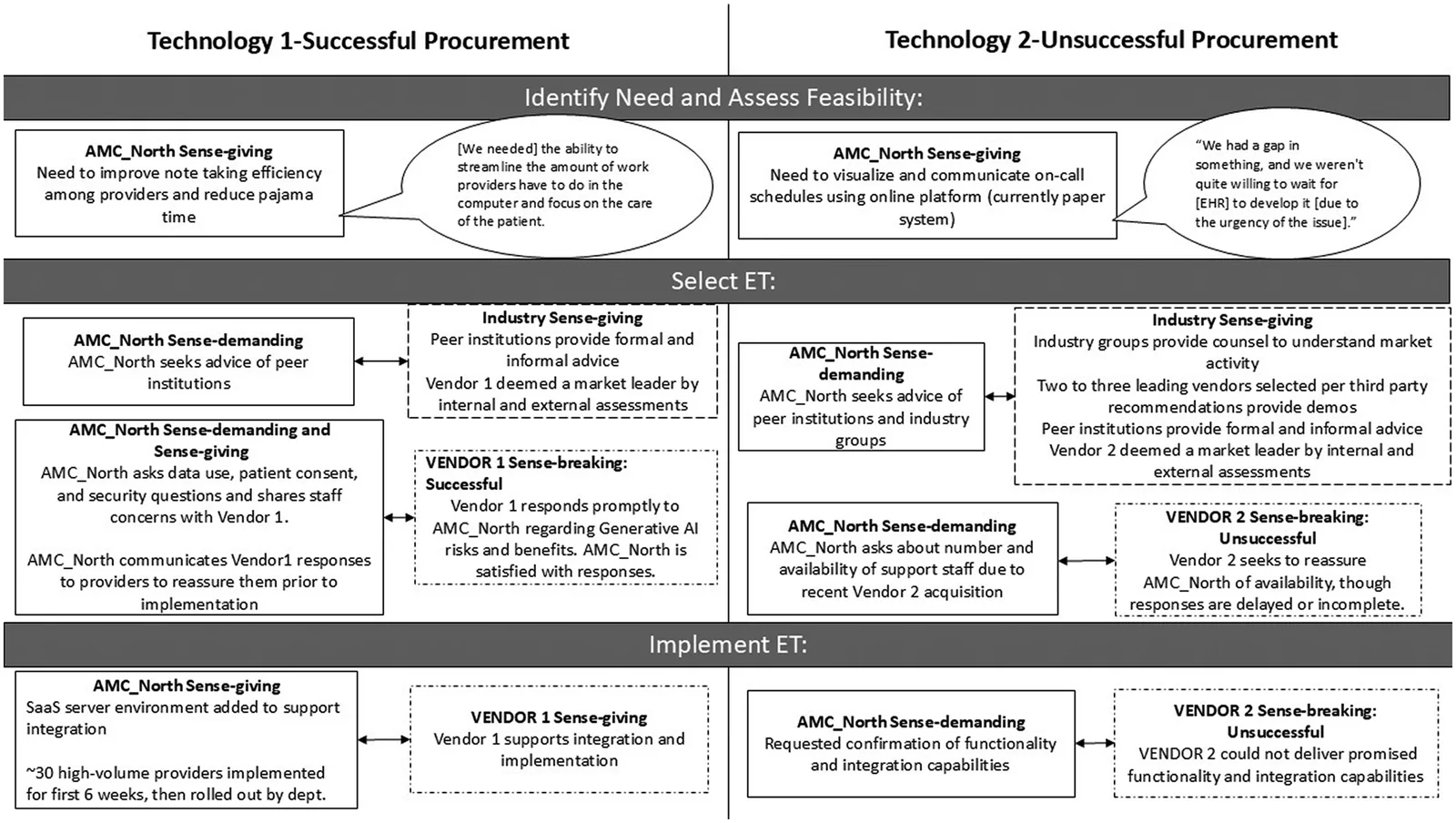
Comparison of sensemaking activities during one successful and one unsuccessful emerging technology procurement and implementation [Source: authors]. Side-by-side comparison flowchart of two electronic technology procurement processes at AMC_North. The left panel, “Technology 1 - Successful Procurement,” shows a need to improve note-taking efficiency and reduce pajama time, followed by peer institution advice, information sharing with Vendor 1, responsive vendor communication, and successful implementation support. The right panel, “Technology 2 - Unsuccessful Procurement,” shows a need for online on-call scheduling, followed by peer and industry consultation, concerns about vendor support after an acquisition, delayed or incomplete vendor responses, and failure to deliver promised functionality and integration capabilities. The diagram is organized into three phases: Identify Need and Assess Feasibility, Select ET, and Implement ET, illustrating how communication and vendor responsiveness contributed to different procurement outcomes.

Table 2 provides illustrative quotes. We identify both the presence of and the absence of the 3 sensemaking components. When a sensemaking activity is engaged by 1 party but not reciprocated by the second party, this is indicative of how sensemaking can help to identify “red flags” earlier in the procurement process.

**Table 2.** Illustrative sensemaking quotes during one successful and one unsuccessful emerging technology procurement and implementation.

	T1 (ambient scribe)	T2 (inter-provider clinical communications)
**Identify need and assess feasibility**	Sense-giving example: “[We needed] the ability to streamline the amount of work providers have to do in the computer and focus on the care of the patient.”	Sense-giving example: “We had a gap in something, and we weren’t quite willing to wait for [EHR] to develop it [due to the urgency of the issue].”
**Select ET**	Sense-demanding example: “A lot of it has to do with the vendor’s market position…and reputation. [vendor 1] is probably the market leader, so we know that the products are probably going to work.” Sense-giving example: “We’ve already started communication with the physicians about how this [is a] large language [model]… The turnaround time is going to be minutes, not hours and the product that we saw, even in just the displays and what I was exposed to, I could already tell that it’s going to be a better product.”	Sense-demanding example: “We went to the market. We looked at a number of different vendors at the time. Different industry groups give us kind of what’s happening in the market… They get…information from [health systems] like us… At the time [vendor 2] was a leader in the space.”
**Implement ET**	Sense-giving example: “As expressed by another participant, “we get a lot of benefit out of the integration between the different tools.”	Sense-breaking example: “They promised a lot of functionality they weren’t able to deliver on…[also, given our EHR’s] market share for large healthcare organizations…everyone out there’s like, ‘Oh, yeah we integrate with that’ and then you try to do it and it doesn’t work.”

#### T1 by vendor 1 pre-implementation

AMC_North sought to improve provider note-taking efficiency and retain clinicians, particularly those with high “pajama time,” ie, note taking time after hours.^19^ One ET considered was T1 from vendor 1. Vendor 1 is a large communications organization founded ∼30 years ago that initially developed speech recognition technologies using natural language processing and now specializes in conversational generative AI solutions that sense, assess, and respond to human language. Vendor 1 works in multiple industries, including healthcare.

Because AMC_North used previous dictation solutions from vendor 1, they leveraged sense-demanding by contacting comparable health systems for feedback on T1. Sense-demanding was also used to surface concerns about Generative AI within T1 to vendor 1, while simultaneously using sense-giving to communicate the risks and benefits back to providers.

#### T1 by vendor 1 implementation

The technical implementation process utilized sense-giving, including justification of the purchase and installation of a SaaS server environment to support T1 as well as interface modifications to support integration with AMC_North’s EHR. Initial clinical implementation focused on ∼30 clinicians with high outpatient volumes; then, the technology was released to 1 outpatient department at a time.

#### T2 by vendor 2 pre-implementation

AMC_North used an inter-provider clinical communications tool from its EHR provider; however, the existing solution left some unmet inter-provider communication needs. They considered waiting for their EHR to develop the technology further, but ultimately chose to seek an external vendor, leveraging sense-demanding. Vendor 2 is a mid-sized company founded ∼18 years ago that uses a cloud-based platform to offer inter-provider clinical communications technologies, including HIPAA compliant secure messaging, real-time on-call scheduling, instant delivery of critical results, alert mobilization, VoIP calling, and analytics. Vendor 2 experienced project management turnover that caused stops and starts with the project as early as the selection phase; sense-demanding efforts by AMC_North were met with limited sense-breaking, that is, vendor 2 was unable to reassure AMC_North of its ability to meet project management staffing needs.

#### T2 by vendor 2 implementation

During the technical implementation process, AMC_North faced additional challenges with sense-demanding, and vendor 2 provided limited sense-breaking. This challenge is not unique to vendor 2 as studies find that companies face barriers to adopting standards-based application programming interfaces.^1^ This limited sense-breaking gives rise to an opportunity during the procurement process to consider earlier iterative testing of the technical integration functionality when possible. After 2 years of investment, the implementation was abandoned in favor of a partial solution that integrated more seamlessly with AMC_North’s EHR.

#### Overall ET procurement strategy

Table 3 provides examples of the sensemaking activities observed across 3 IT Committee meetings. For example, in meeting 1, leads of existing ET programs used sense-giving by reminding the committee of the program aims, progress, and lessons learned from peer institutions. Committee members also leveraged sense-giving during meeting 2 discussions, as they discussed the benefits of transforming workflows through previously procured and new ETs, eg, cognitive computing models. A robust and positive exchange of sense-demanding and sense-breaking was also observed across meetings 2 and 3 regarding the enterprise AI strategy. Committee members used sense-demanding to request more information about AMC_North’s current cloud and data lake capabilities; helpful sense-breaking occurred as a capability gap that could negatively influence future ET implementations was identified and plans to address the gap were discussed. By meeting 3, AMC_North had fully responded to prior sense-breaking by not only strengthening their cloud capabilities to support future AI-based ET implementations, but also by reconfiguring staff and onboarding new advisors to form an internal AI Organization with ET product owners and product managers, a Responsible AI Committee, and an AI Oversight Committee, providing tactical, operational, and strategic guidance respectively. They also highlighted the importance of SMUGLES, smart users for go-live, for their ET implementations. This was a novel concept within AMC_North that was adapted from the concept of super users from EHR implementation planning,^27^ but with a stronger focus on ET understanding as opposed to use frequency.

**Table 3.** Examples of ET sensemaking activities during AMC_North IT committee meetings.

	Sensemaking activities
**Meeting 1**	Sense-giving:
	Reviewed pipeline process data for critical projects Discussed lessons learned from peer institutions
**Meeting 2**	Sense-giving:
	Discussed enterprise AI strategy to use the cloud as the enabler, data as driver of future AI implementations
	Sense-demanding:
	Assessed current Cloud and Data Lake Capabilities to address gaps
	Sense-breaking-Present and robust:
	Current cloud and data lake capabilities insufficient for implementation of some ETs using AI ○ Decision to develop a proof of concept AI sandbox ○ Decision to enhance AI Governance and Operating model and Responsible AI Framework
**Meeting 3**	Sense-giving:
	Decisions from meeting 2 implemented, including:
	Product owners and product managers Responsible AI Committee AI Oversight Committee
Exemplary Quote Motivating ET Strategic Changes from AMC North Executive: “Technology is going to change the way we practice medicine more in the next 5 years than it has in the last 100. And it’s just moving so fast, and there will be winners and losers in healthcare systems, but it’s going to be those that can pick the right thing to move forward with and use the limited resources that we all have to the best of our ability. If you could watch [the IT sourcing] committee work…they say no more than yes. And they have to because there’s a limited pool of resources…[If] you make a mistake, can you spend 4 years on something that costs you $20 million, just to trash it later. And that’s a decision that has a very negative impact.”	

## Key recommendations

Our case study reveals 3 key recommendations for IT-related procurement leaders in health systems, summarized in ET-P framework (Figure 2) that positions sensemaking at the center of need identification, ET selection, and implementation, surrounded by an outer loop of evolving technical and organizational infrastructure. Adapting the approach of periodic reflection for implementation research,^28^ we highlight how each of our recommendations relate to the dynamic implementation ecology, procurement team sensemaking and learning, and adaptions to the implementation plan (specifically infrastructure) that occur during implementation; see also.^29^ Adaptations to the implementation itself (in this case the ET) was less salient in this study, but could be examined in future research.

**Figure 2.**
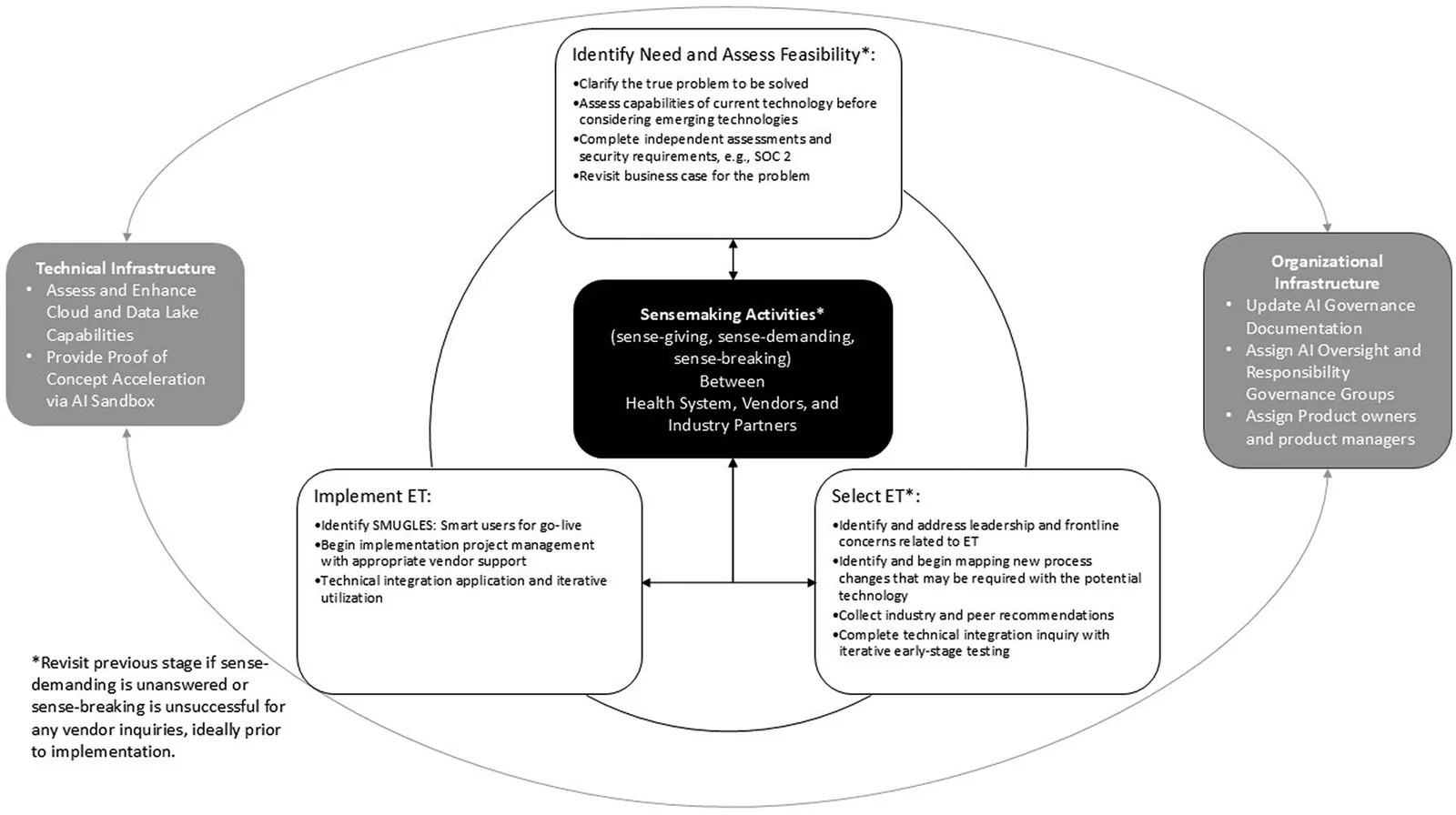
Sensemaking framework for Emerging Technology-Procurement, highlighting key role of sensemaking throughout the procurement process, including identifying need and assessing feasibility, selecting emerging technologies, and implementing emerging technologies, with an emphasis on necessary changes to the technical and organizational infrastructure [Source: Authors].

### Dynamic implementation ecology

Dynamic, Multi-Stakeholder Sensemaking Model. First, health systems should consider replacing linear, internally focused procurement checklists^7^ with a dynamic, multi-stakeholder sensemaking model that more fully accounts for adaptations and interactions in the context over time. In practice, this means formalizing structured information exchanges with ET vendors and industry partners throughout the procurement lifecycle, not only at initial vendor evaluation or contract negotiation. For example, procurement teams can designate liaisons responsible for managing and documenting ongoing sense-giving and sense-demanding exchanges.

### Procurement team sensemaking and learning

Second, procurement should consider intentionally deploying sense-giving, sense-demanding, and sense-breaking across need identification, selection, and implementation, with particular attention to early warning signals when vendors fail to respond effectively to sense-demanding. This can involve the following activities:

1. During need identification, conduct internal sense-demanding to assess whether existing EHR capabilities can address the gap before approaching external vendors.
2. During selection, require structured sense-demanding and sense-breaking from multiple external sources: peer health systems, third-party assessors, and vendors themselves. Treat a vendor’s inability to respond adequately to sense-demanding (eg, on staffing stability or EHR integration readiness) as an early “red flag” that should be noted within ET project documentation.
3. During implementation, consider requesting that potential vendors complete iterative integration testing at defined milestones prior to full deployment,^30^ converting technical sense-demanding into a contractual checkpoint rather than an informal inquiry.

Adaptation of implementation plan, including infrastructure: third, the ET procurement process should not only address the direct need and ET in question, but also yield new infrastructure to support future change. For example, sense-giving and sense-demanding can be used to identify cloud capability gaps while sense-breaking can be used to question the status quo and consider a new course of action, eg, make cloud and data lake capability enhancements. Such technological advancements may also give rise to new organizational infrastructure (AI Oversight and Responsible AI committees) to assess needs, opportunities, and risks.

## Conclusions

This case demonstrates that successful ET implementation in health systems depends not only on technical fit, but on the quality of sensemaking between the health system, ET vendors, and industry partners. As this case is based on 1 health system, future research should examine the application of the sensemaking ET-P framework across additional sites. Future studies should also consider operationalizing our model to create a scoring criterion for sense-giving, sense-demanding, and sense-breaking along each stage of the ET-P framework for healthcare systems.

## Data Availability

Raw qualitative data from this study cannot be shared publicly.
